# Distant functional connectivity for bimanual finger coordination declines with aging: an fMRI and SEM exploration

**DOI:** 10.3389/fnhum.2014.00251

**Published:** 2014-04-25

**Authors:** Sachiko Kiyama, Mitsunobu Kunimi, Tetsuya Iidaka, Toshiharu Nakai

**Affiliations:** ^1^Neuroimaging and Informatics Lab, National Center for Geriatrics and GerontologyOhbu, Japan; ^2^Department of Psychiatry, Graduate School of Medicine, Nagoya UniversityNagoya, Japan

**Keywords:** bimanual finger coordination, aging, functional connectivity, fMRI, SEM, PMd, in-phase, anti-phase

## Abstract

Although bimanual finger coordination is known to decline with aging, it still remains unclear how exactly the neural substrates underlying the coordination differ between young and elderly adults. The present study focused on: (1) characterization of the functional connectivity within the motor association cortex which is required for successful bimanual finger coordination, and (2) to elucidate upon its age-related decline. To address these objectives, we utilized functional magnetic resonance imaging (fMRI) in combination with structural equation modeling (SEM). This allowed us to compare functional connectivity models between young and elderly age groups during a visually guided bimanual finger movement task using both stable in-phase and complex anti-phase modes. Our SEM exploration of functional connectivity revealed significant age-related differences in connections surrounding the PMd in the dominant hemisphere. In the young group who generally displayed accurate behavior, the SEM model for the anti-phase mode exhibited significant connections from the dominant PMd to the non-dominant SPL, and from the dominant PMd to the dominant S1. However, the model for the elderly group's anti-phase mode in which task performance dropped, did not exhibit significant connections within the aforementioned regions. These results suggest that: (1) the dominant PMd acts as an intermediary to invoke intense intra- and inter-hemispheric connectivity with distant regions among the higher motor areas including the dominant S1 and the non-dominant SPL in order to achieve successful bimanual finger coordination, and (2) the distant connectivity among the higher motor areas declines with aging, whereas the local connectivity within the bilateral M1 is enhanced for the complex anti-phase mode. The latter may underlie the elderly's decreased performance in the complex anti-phase mode of the bimanual finger movement task.

## Introduction

In daily activities, it is essential to coordinate one's finger movements in tune with information from the environment. Well-coordinated voluntary movements of bimanual fingers allow for various complex tasks such as grooming, cooking and sewing. Such tasks typically require independent bimanual finger movements involving proper timing while observing the specific objects involved (i.e., visuo-motor coordination). Although young people usually portray skillful finger coordination, elderly often experience difficulties in such tasks (Stelmach et al., 1988; Aoki and Fukuoka, 2010). In addition, reduced hand coordination is known to be correlated with particular degrees of mild cognitive impairment (MCI) (Aggarwal et al., 2006; Buracchio et al., 2010) and Alzheimer's disease (AD) (Verheij et al., 2012). Therefore, an effective detection of age-related decline in finger coordination tasks may offer an important indicator regarding the need to initiate treatment to delay the progression of dementia. Nevertheless, it still remains unclear how the neural substrates for the finger coordination change with aging, especially for complex bimanual finger control.

**Figure 1:**
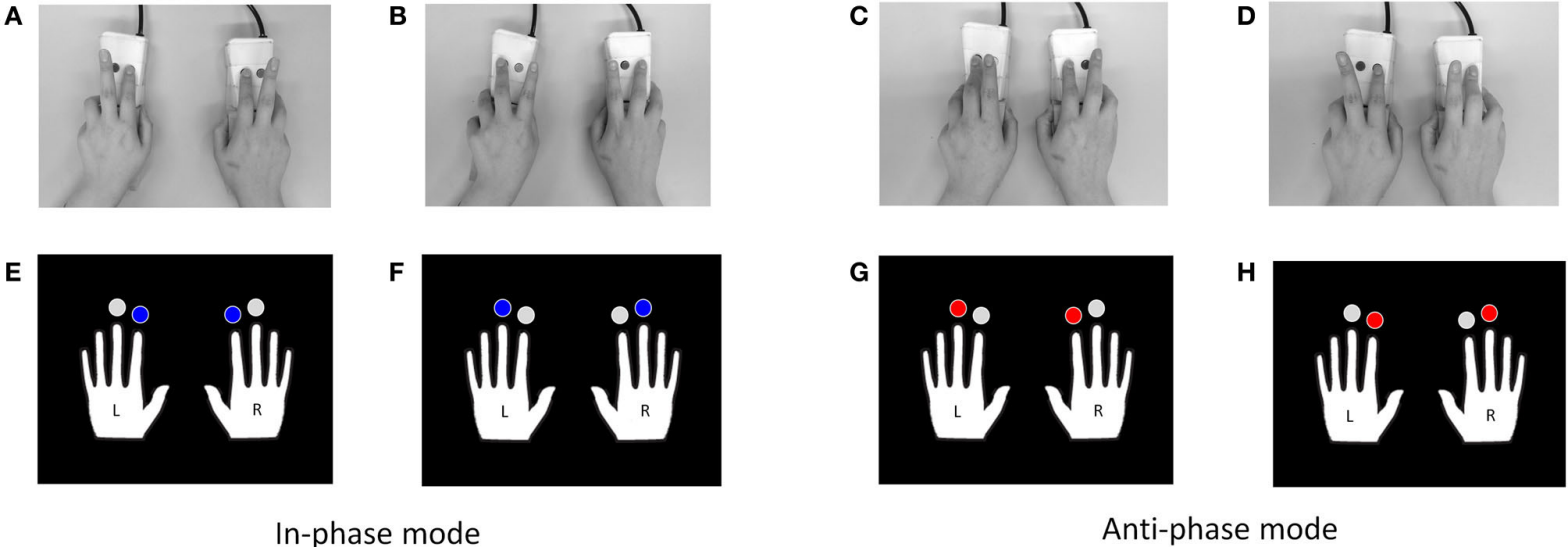
**Examples of the visually-guided bimanual finger movement task**. The task consists of in-phase (alternation of **A** and **B**) and anti-phase (alternation of **C** and **D**) modes using the index and middle fingers of both hands. Two pairs of pictures in the lower rows were used as visual pacing cues for blocks of in-phase (alternating presentation of **E** and **F**) and anti-phase **(G,H)** modes.

The bimanual finger movement task is one of the most common paradigms to investigate voluntary human finger coordination. Our version of the task consisted of alternating the index and middle finger through a visually guided pattern. When performing the task in in-phase (i.e., mirror symmetrical) and anti-phase (i.e., parallel asymmetrical) modes as shown in Figure 1, the in-phase mode using bimanual fingers is inherently more stable than the anti-phase mode. This is mainly because humans innately use limb movement synchrony in order to maintain body balance (Kelso, 1984; Schöner and Kelso, 1988; Byblow et al., 1994; Mechsner et al., 2001; Hu and Newell, 2011). Involuntary phase transitions from the anti- to the in-phase mode occur even in healthy young adults' performance as the movement frequency increases (Kelso, 1984; Byblow et al., 1994; Aramaki et al., 2006). Additionally, in comparison with young adults, elderly adults exhibit instability and increased latency in response to visual pacing cues (Stelmach et al., 1988), suggesting that the ability to handle the complexity of the anti-phase mode significantly changes with aging.

Previous functional neuroimaging studies using primates and humans have demonstrated that fronto-parietal areas are crucial for effective voluntary visually-guided bimanual coordination (Battaglia-Mayer et al., 2001; Rizzolatti and Luppino, 2001; Rowe et al., 2006; Filimon, 2010). Within these areas, the primary motor area (M1) serves to execute the bimanual finger movements (Schieber and Poliakov, 1998; Aoki et al., 2003, 2005; Scott, 2003, 2008; Koeneke et al., 2004; Jaillard et al., 2005; Gao et al., 2008; Madden et al., 2010), while both the supplementary motor area (SMA) and the premotor area (PMA) are considered to be interactively responsible for planning the preparation of the motor actions involved (Deiber et al., 1996; Nakai et al., 2003; Solodkin et al., 2004; Simmonds et al., 2008; Rowe and Siebner, 2012; Hétu et al., 2013). Furthermore, the primary somatosensory area (S1) and superior parietal lobule (SPL) play notable roles especially in higher-order visuo-motor coordination (Debaere et al., 2004; Stoeckel et al., 2004; Nakai et al., 2005; Stanley and Miall, 2007; Granek et al., 2012; Baumgartner et al., 2013). Lesion studies also support the importance of these regions regarding different aspects of bimanual coordination (Tanaka et al., 1996; Stančák et al., 2003; Jaillard et al., 2005). Altogether, integrative abilities of the aforementioned motor association cortex are assumed to play an important role for successful complex bimanual finger coordination.

Neural control of the elderly adults' bimanual movements has also been investigated by Goble et al. (2010) although they focused on wrist (not finger) movements using auditory pacing cues. Their study demonstrated an age-related increase in activity in regions including the SMA and bilateral secondary somatosensory area (S2). Consistent with these findings, Salardini et al.'s (2012) case study of elderly patients with synkinesia (involuntary movement of body parts when voluntary moving other body parts) showed that SMA activation was observed during a manual movement task (e.g., squeezing a sponge), even though they failed in the actual execution of the movement. These studies suggest necessary SMA involvement for manual movements, regardless of whether they succeed in the execution. However, to date, the effects of aging on the involvement of these areas contributing to bimanual finger coordination has not been investigated thoroughly.

Recent investigations utilizing techniques such as structural equation modeling (SEM) (Zhuang et al., 2005; Rowe et al., 2006; Taniwaki et al., 2006; Laird et al., 2008; Walsh et al., 2008) and dynamic causal modeling (DCM) (Grefkes et al., 2008, 2010; Cieslik et al., 2011; Pool et al., 2013; Rheme et al., 2013; Zilverstand et al., 2014) also support the necessity of functional connectivity within the motor association cortex for successful manual control in young adults. The necessity is particularly evident for bimanual finger movements in the stable in-phase mode, but has not yet been observed in the anti-phase mode. Walsh et al. (2008) proposed a connectivity model utilizing regions of interest (ROIs), particularly the M1, S1, SMA, dorsal PMA (PMd) and SPL (all bilateral), which highlights differential roles for the dominant and non-dominant hemispheres. Their SEM models suggested that the intra-hemispheric network within the dominant hemisphere is responsible for initiating movements and, additionally, that the inter-hemispheric connection between the non-dominant to the dominant hemisphere (mainly in the PMd) governs the organization of movements in space and time. It seems therefore clear that both the intra- and inter-hemispheric connections are required to be cooperatively activated for successful bimanual finger movement, specifically within the stable in-phase mode. Accepting this, then, the question arises regarding how these neural networks are realized for the more complex anti-phase mode. The phenomenon of involuntary phase transitions from the anti- to the in-phase mode (Aramaki et al., 2006, 2010, 2011; Kelso, 1984) posits that, because of its tendency to resist human's innate synchrony, the anti-phase mode recruits more intense connectivity within regions associated with coordinating temporal and spatial information of both hands, namely, inter-hemispheric connections within motor associative regions (including the PMd). Consequently, this raises the question whether declined bimanual finger coordination is related to the above-mentioned networks. Although the previous investigations suggesting the importance of intra- and inter-hemispheric connections for bimanual finger movements have targeted young adults, it can be predicted that the elderly's less accurate bimanual finger coordination may derive from any age-related decline in the connectivity.

Taken together, the aims of the present study are (1) to characterize functional connectivity within the motor association cortex required for complex bimanual finger coordination, and (2) to detect any age-related decline in the connectivity. To address these objectives, we utilized fMRI and SEM to compare the functional connectivity models between young and elderly age groups during a visually-guided bimanual finger movement task using the stable in-phase and the complex anti-phase modes.

## Materials and methods

### Participants

Twenty healthy young adults (10 males; age range: 19–39, *M* = 25.2, *SD* = 5.5) and 20 healthy elderly adults (11 males; age range: 61–74, *M* = 68.2, *SD* = 4.0) participated in the fMRI experiment in accordance with the ethical principles stated in the Helsinki Declaration, after giving written informed consent. All the participants were confirmed to be right-handed as they scored greater than 70 for the H. N. Handedness Inventory (Hatta and Hotta, 2008) which is a modified Japanese version of the Edinburgh Handedness Inventory (Oldfield, 1971). We also administered a mini-mental state examination (MMSE; Folstein et al., 1975) to screen for possible cognitive impairment, and a geriatric depression scale (GDS; Sheikh and Yesavage, 1986) to screen for depressive symptoms. All of the participants scored over 26 for the MMSE (Young: *M* = 29.5, *SD* = 0.9; Elderly: *M* = 29.3, *SD* = 1.6) and less than 8 for the GDS (Young: *M* = 2.7, *SD* = 2.2; Elderly: *M* = 1.2, *SD* = 1.6). The study protocol was approved by the Ethics Committee of the National Center for Geriatrics and Gerontology, Japan.

### Behavioral data acquisition and analysis

A visually-guided bimanual finger movement task with stable in-phase and complex anti-phase coupling modes was administered to young and elderly participants. We set visual pacing cues for both modes at three specific levels, namely: 1.0, 1.5 and 2.0 Hz respectively. This was done in order to seek more salient behavioral age-related differences suitable for selection for the fMRI and SEM analyses. Therefore, the design had 2 coupling modes (in- vs. anti-phase) × 3 levels of visual pacing cue frequency (1.0, 1.5 and 2.0 Hz) × 2 age groups (young vs. elderly) layout.

As shown in Figure 1, the in-phase mode refers to the periodic alternation of synchronous pressing of both index fingers (A) and synchronous pressing of both middle fingers (B). The anti-phase mode represents a periodic alternation of synchronous pressing of the left middle and the right index fingers (C), and synchronous pressing of the left index and the right middle fingers (D). For the in-phase mode, a pair of pictures (E and F) depicting the right and left hands, in which blue marks were placed above both index fingers in one picture (E) and above both middle fingers in the other (F), were alternately presented as visual pacing cues. Another pair (G and H) was prepared for the anti-phase mode such that red marks were placed above the left middle and the right index fingers in one picture (G), and above the left index and the right middle fingers in the other (H).

The task employing a blocked design included three runs, each of which consisted of alternating 20s-lasting resting state blocks (9 blocks) and visual cue presentations for the two modes. On each run, four in-phase blocks and another four anti-phase blocks were alternately administered. The three runs differed in the pacing cue frequency. All the participants were presented with cue presentations in the same order (from the lowest to the highest frequency). The total task duration was 5 min and 40 s [20 s × (9 blocks + 8 blocks) = 340 s].

The participants wore VisuaStim digital goggles (Resonance Technology, Inc., Northridge, CA, USA) mounted to the head-coil to view the pacing cues projected in the display at 800 × 600 resolution, while laying supine in the MR scanner. Vision corrective lenses were attached to the goggle if needed. Participants were asked to hold a pair of custom-made wooden joysticks in which the response pads (Model HHSC—2 × 2; Current Designs, Philadelphia, PA, USA) were embedded, and to use their index and middle fingers of both hands to press the four buttons in tune with the visual pacing cue presentation. We used Presentation (Neurobehavioral Systems, Albany, CA, USA) for presenting the pacing cues and obtaining behavioral data.

To ascertain whether bimanual finger movements were correct, regardless of the mode, time differences should be close to zero between bimanual fingers that were required to be pressed synchronously. We subsequently defined movements as correct, depending on whether the button-pressing time difference of a finger of one hand with the paired finger of the other was smaller compared to the unpaired one, as described in Table 1. For instance, a left index finger movement (LI) can be judged as correct when the difference of the LI pressing time with RI (right index finger) is smaller than that of the RM (right middle finger) for the in-phase mode, and vice versa for the anti-phase mode.

**Table 1:** **Definition of correct movements for the bimanual finger movement task**.

**Latency of one hand**		**Difference with the other hand**	
		**Correct in-phase (Same finger < different finger)**	**Correct anti-phase (Different finger < same finger)**
Index finger	RI	LI < LM	LM < LI
	LI	RI < RM	RM < RI
Middle finger	RM	LM < LI	LI < LM
	LM	RM < RI	RI < RM

*Notes: R, right; L, left; I; index; M, middle*.

The behavioral accuracy rate was calculated by dividing the total number of the correct movements by the total number of button-presses per condition per participant. A 2 (i.e., in- and anti-phase modes) × 3 (i.e., pacing frequencies at 1.0, 1.5, 2.0 Hz) × 2 (young and elderly age group) repeated measures ANOVA was conducted in which the mode and pacing frequency were treated as within-participant factors, and age as a between-participant factor. PASW statistics 18J (SPSS, Chicago, IL, USA) was used for the analysis.

### fMRI data acquisition and analysis

The magnetic resonance (MR) images were acquired on a 3T MR scanner (Siemens Trio, Erlangen, Germany) with a Siemens 12-channel head coil. T2^*^ weighted gradient echo planar imaging (GRE-EPI) sequences were acquired with the following parameters: *TE* = 30 ms, *TR* = 3000 ms, flip angle = 90°, matrix 64 × 64, field of view = 192 mm, 39 axial slices, slice thickness = 3 mm, and distance factor = 25%. For each EPI run, 113 volumes were acquired. A three-dimensional MPRAGE high-resolution T1-weighted image (*TR* = 2530 ms, *TE* = 2.64 ms, flip angle = 7°, matrix 256 × 256, FoV = 250 mm, 208 slices per slab, slice thickness = 1 mm, and distance factor = 50%) was also acquired for anatomical details.

The functional images were preprocessed and analyzed with SPM 8 (Wellcome Department of Cognitive Neurology, London, UK) implemented in Matlab (Mathworks, Sherborn, MA, USA). Functional images were first resliced and subsequently realigned with the first scan as a reference to correct for head movement. We then co-registered the T1-anatomical image to the first functional image. The co-registered image was used to create a template using a diffeomorphic flow-based algorithm approach (DARTEL; Ashburner, 2007). Subsequently, all functional images were normalized to the template image as defined by the standard Montreal Neurological Institute (MNI). Finally, we smoothed the spatially normalized functional images with an isotopic Gaussian kernel of 6 mm full-width at half-maximum (FWHM).

After the preprocessing procedure, analysis for each participant was conducted on the basis of the general linear model (GLM; Friston et al., 1995) to extract contrasts between rest and each of the conditions of the task (i.e., 2 mode types × 3 pacing levels). At the first level, each event was modeled as a hemodynamic response function (HRF) with its temporal derivative. At the second-level analysis, the results for each participant were entered into the random effects model by using 1-sample *t*-tests to obtain the contrast images of parametric maps of each age group. The threshold was set at significance level of *p* = 0.05 with family-wise error (FWE) correction. The spatial extent threshold was set at *k* = 30 voxels.

### Functional connectivity analysis with structural equation modeling (SEM)

The aim of this analysis was to explore how different brain regions within the motor association cortex interact with each other during the bimanual finger movement task, and to demonstrate how the interaction depends on young and elderly age groups in the in- and anti-phase modes. SEM, along with DCM, is one of the most widely used approaches to model neural connectivity between multiple ROIs extracted from fMRI data (McIntosh and Gonzalez-Lima, 1994; Lindquist, 2008). Using Bayesian model estimation, DCM can include a large set of parameters such as HRF, resting connection strength between regions, and beta weights concerning the amount of influence from the experimental manipulations (Friston et al., 2003, 2011), however, it lacks an adequate way to evaluate model fit (Lohmann et al., 2012). SEM based on measures of covariance, conversely, provides many indices to estimate model fit (Büchel and Friston, 1997). However, specific neural characteristics as found in DCM are not standard taken into account since SEM is not a specific technique for neuroimaging research. As such, DCM is a highly model-dependent method in which its pre-specified model has to be evaluated to have good fit, while SEM is a model-free, exploratory technique with essentially unrestricted freedom to assume and modify models until the best solution is achieved. Given the present exploratory study's objective to detect age-related changes in the functional connectivity for bimanual finger coordination (for which the exact parameters are unspecified), we chose to employ SEM, instead of DCM, to better model the functional connectivity within the motor association cortex during the bimanual finger movement task.

SEM estimates causal relationships on the basis of path coefficients between multiple variables. The variables used in SEM can be considered both observed and latent (i.e., unobserved). Since analyses of neural activity with fMRI use blood oxygenation level-dependent (BOLD) signals observed from the brain, the dependent measures should be treated as observed variables, instead of latent ones (McIntosh et al., 1994). As noted above, one advantage of SEM is that it evaluates goodness-of-fit between the obtained data and the predicted model. Even if some path coefficients are found to be significant, the assumed model cannot be statistically accepted unless goodness-of-fit indices meet the conventional criteria, which support the robustness of the model as a whole.

The procedure adopted in our SEM assessment examined using AMOS 21J (SPSS, Chicago, IL, USA) with the maximum likelihood estimation method was the following:

(1) As a first step, following Walsh et al. (2008), ROIs were restricted to five areas in the motor association cortex: M1, SMA, PMd, S1, and SPL (all bilateral). We then specified peak coordinates within each ROI resulting from a conjunction analysis of all four contrasts, which were subsequently used these to extract the ROIs in the four individual contrasts of each mode (in- and anti-phase) for each age group (young and elderly) in comparison with rest.

Next, (2) among the above-mentioned ROIs, four causal models were postulated for each of the young and elderly groups for both the in- and anti-phase modes by drawing paths between multiple variables within a model. Parameter estimates of the ROIs were extracted and adopted as the observed variables of the model. Our model evaluation started by applying Walsh et al.'s (2008) path-drawing procedure (Walsh et al., 2008; Model C, Figure 4, p. 547) which solved the functional connectivity for the young adults' in-phase bimanual finger movements using the thumbs and index fingers.

Next, (3) SEM sought parameters which minimized the difference of the covariance structures between the predicted model and the given data. Whether or not the model fitted the data was tested by the *chi-square* (χ^2^) statistic, of which the non-significance supports the homogeneity (i.e., supports fit). Besides the *chi-square* test, the goodness of fit is evaluated by other indices such as the goodness-of-fit index (GFI) and the comparative fit index (CFI) which are conventionally required to exceed 0.95. Additionally, the root mean square error of approximation (RMSEA) needs to be less than 0.05 (Schumacker and Lomax, 2004).

However, (4) since Walsh et al.'s SEM model assumption (i.e., their path drawing approach) did not fit our data according to the goodness-of-fit indices obtained from the SEM, several path drawings were modified and then re-evaluated iteratively until the model with best fit was obtained. In order to select a more suitable model among multiple candidate models, the Akaike information criterion (AIC) and Bayesian information criterion (BIC) were evaluated as they are indices for direct model comparison. Not their absolute values, but their relative differences indicate a better fit (the smaller, the better; Schumacker and Lomax, 2004).

Next, (5) Once models employing the best solution were attained for each of the two modes (in- and anti-phase) for both groups (young and elderly), we sought significant standardized path (partial regression) coefficients (β) from one region to another. When comparing a path coefficient differences within the same pathways across tasks or across groups, the difference in magnitude suggests a quantitative change in the functional influence. Additionally, if the sign of the coefficient is different across tasks/groups; it should be interpreted as a qualitative change (McIntosh and Gonzalez-Lima, 1994).

## Results

### Behavioral data

**Figure 2:**
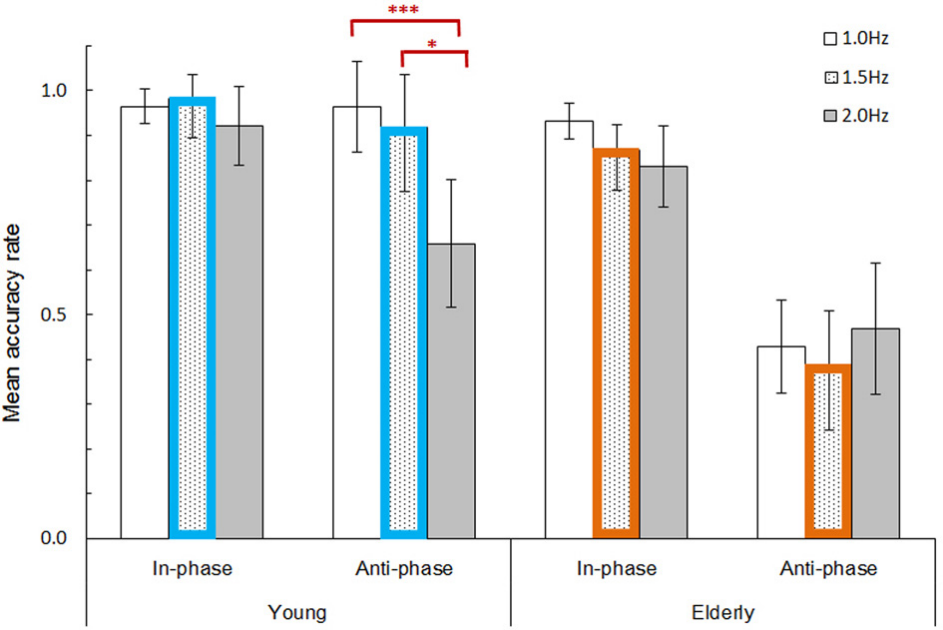
**Mean behavioral accuracy rate (correct movements / total button-presses) divided by mode (in- and anti-phase), pacing cue frequency (1.0, 1.5, and 2.0 Hz), and age group (young and elderly)**. Error bars represent 95% confidence intervals. Bar plots with colored borders (i.e., 1.5 Hz pacing frequency; blue for young, orange for elderly) denote the conditions selected for fMRI and SEM analyses. An ANOVA combined with Bonferroni *post-hoc* tests revealed significant differences among the three pacing frequencies for the young group's anti-phase mode. ^*^p < 0.05; ^***^p < 0.001.

Figure 2 shows the behavioral accuracy rates (i.e., number of correct movements divided by the total number of button-presses). A repeated measures ANOVA revealed significant main effects of mode [*F*_(1, 39)_ = 71.944, *p* < 0.001, η^2^_*p*_ = 0.648], pacing frequency [*F*_(2, 78)_ = 8.510, *p* < 0.001, η^2^_*p*_ = 0.179] and age group [*F*_(1, 39)_ = 34.486, *p* < 0.001, η^2^_*p*_ = 0.469]. Significant interactions were found between mode × age group [*F*_(1, 39)_ = 26.508, *p* < 0.001, η^2^_*p*_ = 0.405], pacing frequency × age group [*F*_(2, 78)_ = 7.176, *p* < 0.01, η^2^_*p*_ = 0.155], and a 3-way interaction between mode × pacing frequency × age group [*F*_(2, 78)_ = 7.374, *p* < 0.01, η^2^_*p*_ = 0.159]. Further *post-hoc* comparisons using the Bonferroni correction detected significant differences among the three frequency levels in the young group's anti-phase mode, particularly between the lowest 1.0 Hz (*M* = 96.4%) and the highest 2.0 Hz (*M* = 65.8%, *p* < 0.001), and the moderate 1.5 Hz (*M* = 91.8%) and the highest 2.0 Hz (*p* < 0.05) frequencies.

The overall results revealed that the young group achieved a higher accuracy compared to the elderly group. The elderly group exhibited less accurate behavior particularly in the anti-phase mode throughout all frequency levels. Conversely, the young group accurately performed the anti-phase mode with the lowest 1.0 Hz and moderate 1.5 Hz pacing frequencies, but did not with the highest 2.0 Hz. In other words, 1.5 Hz was the highest pacing frequency (with the anti-phase mode) which was manageable for young participants. The most salient age difference concerning movement accuracy thus can be found at the 1.5 Hz pacing frequency for the anti-phase mode (*M* = 91.8% for young, *M* = 38.7% for elderly), while both age groups showed relatively high accuracy for the in-phase mode (*M* = 98.3% for young, *M* = 86.8% for elderly). To maximize the divergence between task performances, the 1.5 Hz pacing frequency (including in- and anti-phase modes) was selected for subsequent fMRI and SEM analyses.

### fMRI data

**Figure 3:**
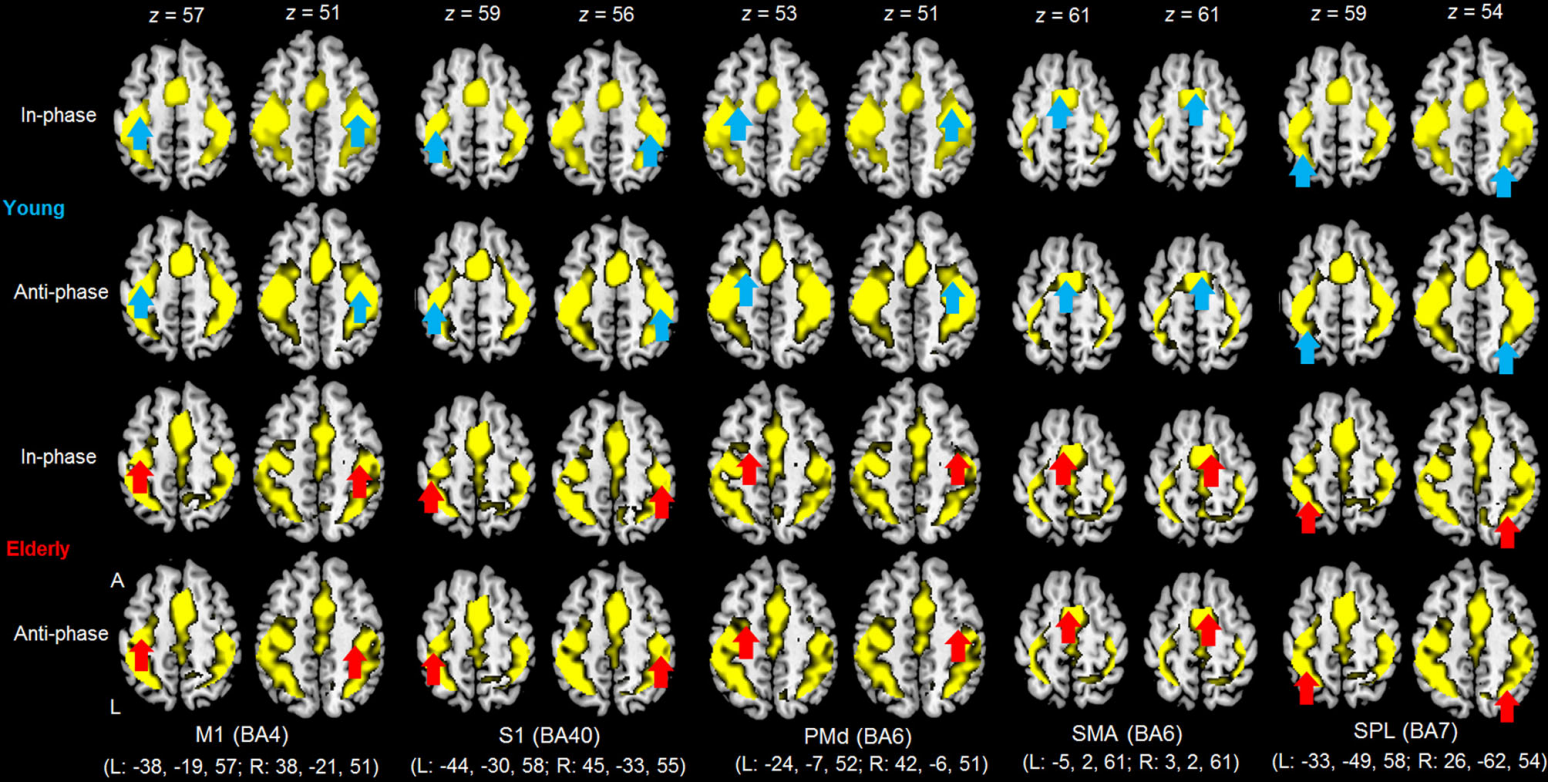
**Regions of interest (ROIs) applied to SEM**. The maps are presented for each mode (in- and anti-phase) for each age group (young and elderly) using a 1.5 Hz visual pacing cues in comparison with rest (group analyses). *p* < 0.05 FWE corrected, *k* = 30 voxels (*n* = 20 for young group, *n* = 20 for elderly group). The ROIs were defined by means of peak coordinates extracted from a conjunction analysis of all four contrasts. M1, primary motor cortex; S1, primary somatosensory cortex; PMd, dorsal premotor cortex; SMA, supplementary motor area, SPL; superior parietal lobule, BA, broadman area; L, left; A, anterior.

As shown in Tables 2 and 3 along with Figure 3, group analyses revealed consistent significant neural activity for the elderly and young age groups during the in- and anti-phase modes of the bimanual finger movement task at a 1.5 Hz pacing frequency at the threshold of *p* < 0.05 with FWE correction, *k* > 30 voxels. Besides the cerebellum and the visual cortex, broad regions in the front-parietal areas including the precentral gyrus, postcentral gyrus, medial frontal gyrus, inferior parietal lobule, and SPL were consistently activated throughout the four rest vs. mode × group contrasts. For the young group contrasts between the anti-phase > in-phase modes revealed greater activation in the right sub-gyral and the right inferior parietal lobule for the anti-phase mode compared to the in-phase mode. The elderly group, on the other hand, did not yield the significant difference between these two modes. Additionally, no significant changes were found in contrasts between the two age groups (both contrasts of elderly > young and young > elderly).

**Table 2:** **Activation regions during the bimanual finger movement task: young group**.

**Region**	**Hem**.	**BA**	**Cluster**	**MNI coordinates**			***t***
			**(Voxels)**	***x***	***y***	***z***	
**IN-PHASE MODE > REST**							
Sub-gyral	R	–	2098	36	−21	48	12.90
Precentral gyrus	L	4	2355	−36	−18	52	10.82
Culmen	L	–	1409	−11	−51	−24	8.38
Medial frontal gyrus	Mid.	6	598	3	2	61	7.97
Middle occipital gyrus	R	37	408	48	−67	4	7.78
Middle temporal gyrus	L	–	213	−48	−69	6	6.58
Sub-gyral	R	–	125	33	−39	49	5.93
Inferior occipital gyrus	R	–	38	32	−88	0	5.48
**ANTI-PHASE MODE > REST**							
Sub-gyral	R	–	4013	36	−21	49	12.53
Postcentral gyrus	L	3	2797	−35	−24	57	10.57
Medial frontal gyrus	Mid.	6	1340	3	2	61	10.02
Dentate	L	–	1726	−12	−51	−24	8.41
Middle frontal gyrus	R	6	46	57	9	36	6.55
Middle occipital gyrus	R	37	97	48	−67	4	6.31
Thalamus	R	–	61	15	−18	−2	6.13
Inferior parietal lobule	R	–	92	58	−28	31	5.77
**ANTI- > IN-PHASE**							
Sub-gyral	R	6	155	23	2	54	6.82
Inferior parietal lobule	R	40	40	43	−37	52	5.80

*Notes: All clusters are significant at p < 0.05 with FWE correction, k > 30 voxels. n = 20. Hem., hemisphere; BA, Broadman area; L, left; R, right*.

**Table 3:** **Activation regions during the bimanual finger movement task: elderly group**.

**Region**	**Hem**.	**BA**	**Cluster**	**MNI coordinates**			***t***
			**(Voxels)**	***x***	***y***	***z***	
**IN-PHASE MODE > REST**							
Medial frontal gyrus	Mid	6	462	2	0	63	7.82
Culmen	R	–	2335	9	−55	−17	7.78
Precentral gyrus	L	4	209	−38	−19	57	7.67
Postcentral gyrus	R	3	269	39	−24	54	7.13
Inferior parietal lobule	R	40	224	62	−28	28	6.81
Inferior parietal lobule	L	40	393	−36	−43	54	6.67
Superior parietal lobule	R	7	50	27	−63	52	6.33
Inferior frontal gyrus	R	9	83	51	9	30	6.29
Precentral gyrus	R	6	35	63	9	27	5.69
Postcentral gyrus	L	3	31	−57	−19	43	5.64
**ANTI-PHASE MODE > REST**							
Culmen	R	–	2346	21	−51	−24	8.07
Postcentral gyrus	L	3	182	−39	−19	57	7.37
Medial frontal gyrus	Mid	6	367	2	0	61	7.17
Postcentral gyrus	R	3	178	39	−24	54	6.67
Middle temporal gyrus	R	–	90	45	−61	10	6.54
Inferior parietal lobule	R	–	137	66	−25	24	6.52
Inferior frontal gyrus	R	9	68	51	9	30	6.37
Superior parietal lobule	R	7	41	27	−63	52	6.26
Inferior frontal gyrus	R	–	107	62	11	27	6.22
Inferior parietal lobule	L	–	268	−35	−42	55	6.16
Postcentral gyrus	L	2	33	−59	−21	39	6.12
**ANTI- > IN-PHASE**							
No supra-threshold activation							

*Notes: All clusters are significant at p < 0.05 with FWE correction, k > 30 voxels. n = 20. Hem., hemisphere; BA, Broadman area; L, left; R, right*.

### Functional connectivity

**Figure 4:**
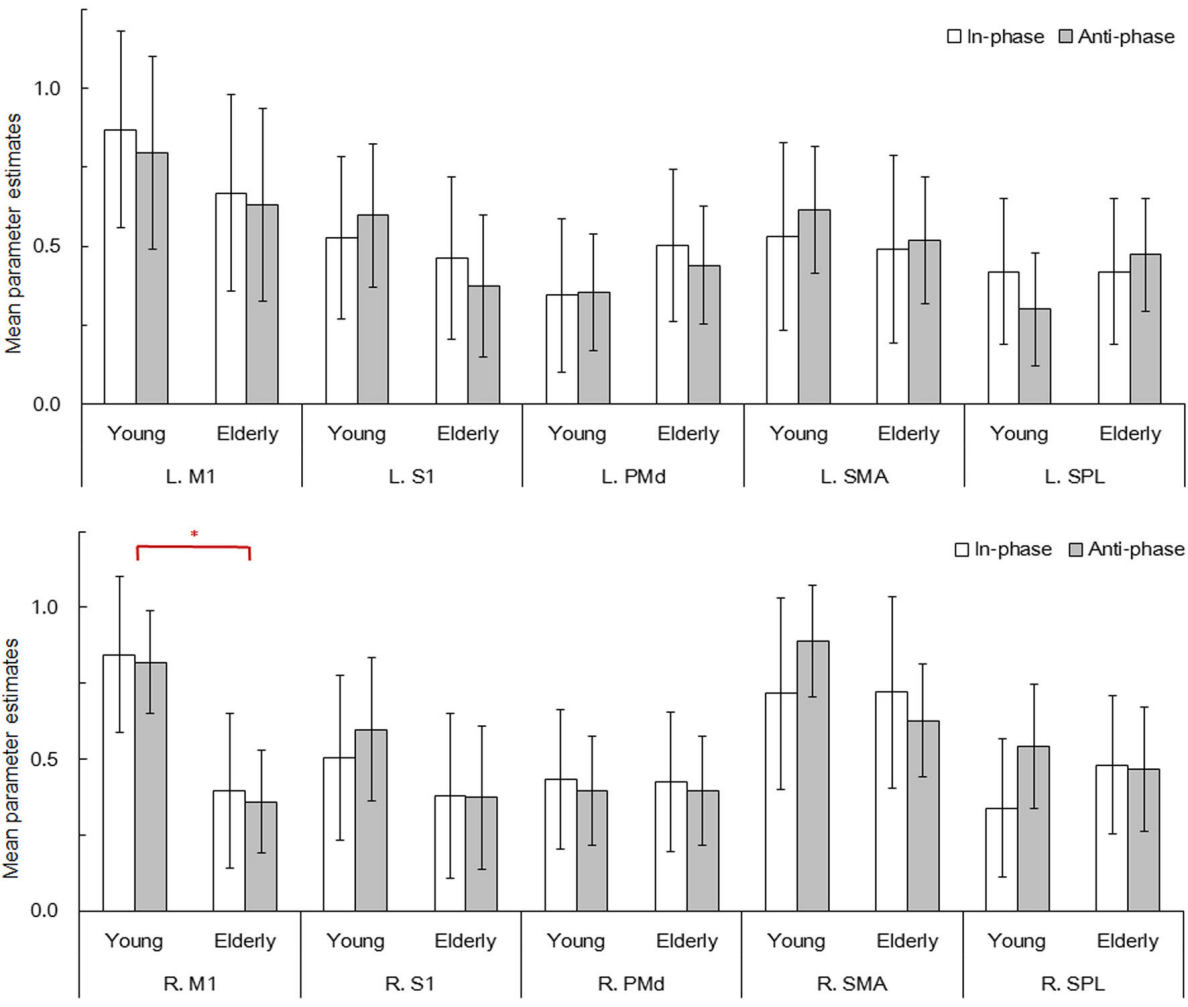
**Mean parameter estimates for ten ROIs during the bimanual finger movement task**. Bar plots represent the movement mode (in- and anti-phase) at a 1.5 Hz visual pacing cue frequency for each of the young (*n* = 20) and elderly (*n* = 20) groups. Error bars represent 95% confidence intervals. M1, primary motor cortex; S1, primary somatosensory cortex; PMd, dorsal premotor cortex; SMA, supplementary motor area, SPL; superior parietal lobule, L; left, R; right. ^*^p < 0.05.

The peak coordinates of the aforementioned ten ROIs (resulting from the conjunction analysis of the four contrasts) are specified in Figure 3 (in MNI coordinates). The parameter estimates of the BOLD signals within the ten ROIs were extracted from the individual contrast images of rest vs. mode × group (as shown in Figure 4). The signal strength differences for each ROI amongst conditions were compared using 2-way repeated-measures ANOVAs (with mode as a within-, and age group as a between-participants factor). Significant age differences were found only in the right M1 (*F*_(1, 158)_ = 6.740, *p* < 0.05, η^2^_*p*_ = 0.041) whereby the young group showed greater activation compared to the elderly group.

**Figure 5:**
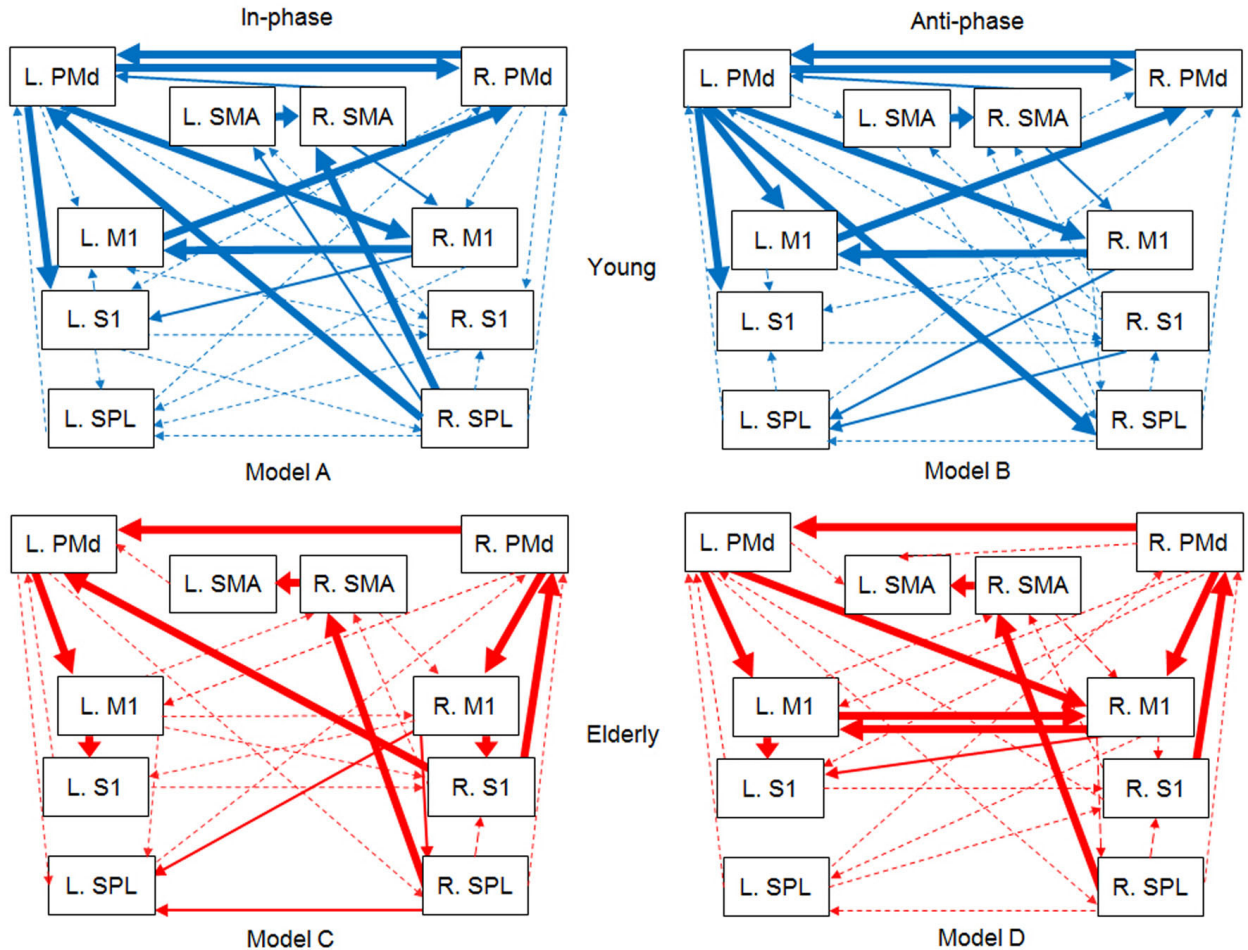
**Functional connectivity models between the ten ROIs within the motor association cortex during the bimanual finger movement task**. The models are presented for each condition of the in-phase (model **A**) and anti-phase (model **B**) modes for the young group, and the in-phase (model **C**) and anti-phase (model **D**) modes for the elderly group. The ROIs applied to SEM as observed variables are presented in rectangles. The dependent measure was parameter estimate obtained from each ROI. Measurement error terms are not shown. M1, primary motor cortex; S1, primary somatosensory cortex; PMd, dorsal premotor cortex; SMA, supplementary motor area, SPL; superior parietal lobule, L; left, R; right. The thickness of the arrows represents the significance levels of standardized path coefficients (β) among the mean parameter estimates of the ROIs as follows: 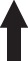
*p* < 0.001; 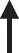
*p* < 0.01; 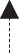
*p* ≥ 0.01.

The SEM result is graphically represented in Figure 5 as a path model illustration for each condition. The four models were confirmed to have a good fit with each condition of the current data, as all of the goodness-of-fit indices met the conventional criteria (Table 4). The standardized path coefficients (β) obtained from SEM for the four models are denoted in the Appendix as Supplementary Tables 1 (for the young group models A and B) and 2 (for the elderly group models C and D).

**Table 4:** **Goodness-of-fit indices obtained from SEM which evaluate the models for functional connectivity during the bimanual finger movement task**.

**Model**	**Group**	**Mode**	***Chi-square* test**	**GFI**	**CFI**	**RMSEA**
A	Young	In-phase	χ^2^_19_ = 8.686,	0.978	1.000	0.000
			*p* = 0.978, *ns*.			
B		Anti-phase	χ^2^_19_ = 17.588,	0.957	1.000	0.000
			*p* = 0.550, *ns*.			
C	Elderly	In-phase	χ^2^_19_ = 16.090,	0.963	1.000	0.000
			*p* = 0.653, *ns*.			
D		Anti-phase	χ^2^_19_ = 17.142,	0.961	1.000	0.000
			*p* = 0.580, *ns*.			

Notes: Models A to D refer to those presented in Figure 5. Result of chi-square tests should be non-significant validating the homogeneity (i.e., fit) between the model and the data. GFI, goodness-of-fit index; CFI, comparative fit index; RMSEA, root mean square error of approximation. The first two indices are conventionally required to be greater than 0.95, while the last one to be less than 0.05.

#### Young group models

The path models obtained from the young group (Models A and B, Figure 5) revealed functional connectivity similarities and differences between the in-phase and anti-phase modes. Both modes shared a strong connectivity (i.e., significant standardized path coefficients (β) at significance level of *p* < 0.001 as shown by the thickest arrows in Figure 5) between the bilateral PMd; from the left to the right SMA, from the right to the left M1, from the left PMd to the right M1, from the left PMd to the left S1, and from the left M1 to the right PMd. Additionally, a strong path (*p* < 0.001) was also shared between the left PMd and the right SPL, although the two modes showed the opposite direction. Contrarily, the in-phase mode (Model A) exhibited a strong connectivity (*p* < 0.001) from the right SPL to the right SMA. However, for this path the anti-phase mode (Model B) did not show any significant connectivity. Instead, the anti-phase mode showed a stronger path (*p* < 0.001) from the left PMd to the left M1.

#### Elderly group models

Strong paths (*p* < 0.001) from the right to the left SMA, and from the right to the left PMd were found for both models of the in-phase (Model C) and anti-phase (Model D) modes in the elderly group. The significances were consistent with the young group models. Unlike the young group, both modes in the elderly group shared strong paths (*p* < 0.001) from the PMd to the S1 (via the M1) in the left hemisphere, from the right PMd to the right M1, and from the right SPL to the right SMA, and from the right S1 to the right PMd. Differences between the two modes were also found as follows: The in-phase mode exhibited stronger paths (*p* < 0.001) from the right S1 to the left PMd, and from the right M1 to the right S1 compared to the anti-phase mode. In the anti-phase mode, on the other hand, stronger paths (*p* < 0.001) were found between the bilateral M1, and from the left PMd to the right M1 in comparison with the in-phase mode.

## Discussion

Previous neuroimaging studies concerning functional connectivity (Zhuang et al., 2005; Rowe et al., 2006; Taniwaki et al., 2006; Grefkes et al., 2008; Walsh et al., 2008) have provided informative insights into the neural substrates involved in bimanual finger coordination, however, they have solely targeted young adults. The present study attempted to ascertain whether an age-related decline in functional connectivity for complex bimanual finger movements exists by comparing the stable in-phase and complex anti-phase modes between young and elderly adults. Behavioral and neuroimaging data revealed important differences between the two age groups.

### Behavioral evidence concerning the bimanual finger movement task

Behavioral data revealed that elderly adults, compared to young adults, generally exhibited lower accuracy rates, corroborating with previous studies (Stelmach et al., 1988; Schlaghecken and Maylor, 2005; Aoki and Fukuoka, 2010; Salardini et al., 2012). Elderly adults show fundamental difficulties concerning the voluntary coordination of bimanual fingers particularly within the anti-phase mode, regardless of the pacing cue frequency. This suggests that elderly are more likely to have the involuntary phase transitions from the complex anti-phase mode to the stable in-phase mode (Byblow et al., 1994; Aramaki et al., 2006). Contrastingly, young adults were able to handle the anti-phase mode well for lower pacing frequencies (1.0 and 1.5 Hz), but not for the highest frequency (2.0 Hz). This result agrees well with Aramaki et al.' (2006) phase transition study. As for the elderly's motor control, Stelmach et al. (1988) observed greater asynchrony in the initiation of bimanual movements. Likewise, Schlaghecken and Maylor (2005) reported an age-related decline in initiating movements after particular intervals. Therefore, our elderly participants' vulnerability to the phase transition shown in the present behavioral data may (at least partially) derive from particular difficulties controlling movement initiation.

### Neural activity within the motor association cortex for bimanual finger movements

fMRI analyses for elderly and young participants indicated neural activation in a broad range of the motor association cortex during the task (see also: Battaglia-Mayer et al., 2001; Rizzolatti and Luppino, 2001; Rowe et al., 2006; Filimon, 2010). The present study consequently specified ten ROIs following Walsh et al. (2008). Among these ROIs which were extracted from the result of a conjunction analysis of four conditions (the in-phase and anti-phase modes in young and elderly groups), ANOVAs indicated age-related differences only in the right M1, whereas those in the remaining ROIs showed no significant differences between the two age groups. The elderly group, relative to the young group, showed decreased activation in the right M1 for both the in-phase and the anti-phase modes, whereas activation in the left M1 was maintained. Given the observation that the elderly participants were less apt in performing the bimanual finger movement task, age-related issues may underlie the imbalanced activation within the bilateral M1 which in turn could influence the movement execution.

### What kind of functional connectivity is required for accurate bimanual finger movements?

In the present study, a model-generating strategy using SEM was applied to ascertain the functional connectivity underlying the bimanual finger movement task. Models in the in-phase and anti-phase modes by the adequately performing young group suggest functional connectivity required for succeeding in bimanual finger coordination in accordance with visual information. The current young group models also exhibited significant connections pertaining to all selected ROIs within the motor association cortex. Specifically, the young group's model in both the in-phase and the anti-phase modes shared strong inter-hemispheric connections between the bilateral PMd, the bilateral SMA, the bilateral M1, the left PMd and the right M1, the left M1 and the right PMd, and the left PMd and the right SPL. This result supports previous investigations concerning bimanual coordination (Zhuang et al., 2005; Grefkes et al., 2008; Walsh et al., 2008) which also suggest inter-hemispheric connections. The present SEM investigation provides compelling evidence that diverse regions within the motor association cortex are recruited to create intense inter-hemispheric connections to allow for complex bimanual finger movements.

It should also be noted that intra-hemispheric connectivity within the left (dominant) hemisphere is increased when comparing the young group's anti-phase mode with the in-phase mode. The young group's SEM results indicate that the connection strength starting from the PMd is increased toward the M1 in the dominant hemisphere for the anti-phase mode, compared to the in-phase mode. Importantly, Walsh et al's. (2008) SEM investigation focusing on the in-phase mode showed that the dominant intra-hemispheric network results in dominant hand movements preceding non-dominant hand movements. Our assessment of the in-phase and anti-phase modes extends these findings such that the precedence of the dominant hand is strongly expected within the anti-phase mode, in which more refined monitoring is required to resolve time differences between the dominant and non-dominant finger movements.

Overall, the present findings of intra- and inter-hemispheric connectivity surrounding the left PMd implies that the PMd may act as an intermediary station within the motor association cortex of the dominant hemisphere. The PMd is considered to be responsible for predicting the consequences of the current motor plan, and for optimizing the plan in order to correct inaccuracies (Churchland et al., 2006). The anti-phase mode requires efforts to carefully monitor the movement's consequences and to avoid instabilities, since it resists the human innate tendency for synchronized bilateral movement. The present study contributed to this discussion by showing that the PMd in the dominant hemisphere has a crucial role for right-handers to inhibit the involuntary phase transition.

### Aging effects on functional connectivity for bimanual finger coordination

The present data revealed specific aging effects, particularly the observation that some connections are maintained while others decline. We found that a strong connection between the dominant (right) and non-dominant (left) SMA was shared by all the four models (both in- and anti-phase modes for both age groups; see also Aramaki et al., 2010, 2011). This is not unexpected as the necessity of SMA-related connectivity has been observed by previous SEM explorations on manual movements by young adults (e.g., Zhuang et al., 2005; Grefkes et al., 2008; Walsh et al., 2008; Cieslik et al., 2011). Specifically, Walsh et al. (2008) have demonstrated that the connection from dominant to non-dominant SMA drives movements for the non-dominant hand. Importantly, the present findings suggest that the SMA indeed works as the principal coordinator for bimanual finger movements, regardless of mode type and age.

Another similarity between the young and elderly groups was found for the connectivity within the bilateral PMd. Along with the young group, the elderly group exhibited strong connections between the bilateral PMd, even though the paths in the elderly group were unidirectional. This suggests that local connections within the PMd are usually preserved with typical aging, even though their strength may somewhat weaken. The PMd connectivity is not just restricted within the region itself, but also extends to diverse regions among the motor association cortex. As discussed earlier for the young group models, the strong intra- and inter-hemispheric connections surrounding the PMd for the dominant hemisphere appear to play a principal role in successfully executing complex bimanual finger movements. However, the elderly group did not recruit these strong connections as fully as the young group did. In the elderly group's anti-phase mode, unlike the young group, paths from the dominant PMd to the non-dominant SPL, and from the dominant PMd and to the dominant S1 were statistically not significant. Therefore, an age-related decline can be clearly observed within the intra- and inter-hemispheric connectivity between the dominant PMd and other distant regions, rather than adjacent regions.

As noted earlier, the PMd is responsible for monitoring and optimizing motor action. Furthermore, the SPL seems to be involved in mismatch recognition between visual cues and bimanual hand movements (Stoeckel et al., 2004; Baumgartner et al., 2013), while the S1 plays a key role in sustaining a high synchronization level between hand movements (Bourguignon et al., 2012). Sufficient intra- and inter-hemispheric connections among these distant regions within the higher motor areas appear to be especially important for accurate bimanual finger coordination. Our findings concerning age-related changes within the dominant PMd also generally agree with Rowe et al.'s (2006) study on the human motor system (using positron emission tomography and transcranial magnetic stimulation). Rowe et al. reported that local connectivity within the bilateral PMd was enhanced while its connectivity with remote regions was declined in healthy elderly adults. The present exploration adds to Rowe et al.'s findings as age-related decline in functional connectivity for motor coordination does not occur concurrently within the whole motor association cortex, but begins within distant regions surrounding the PMd for the dominant hemisphere.

Especially for the elderly adults' anti-phase mode, we found that the connections within the bilateral M1 were enhanced, whereas distant connections among the higher motor areas were decreased. These age-related changes might underlie the elderly's poor performance concerning the fairly demanding anti-phase mode (in which much more attention is required to avoid the involuntary phase transition to the more stable in-phase mode). This suggests that elderly adults have difficulties with the motor planning and monitoring stages, which are mainly controlled by the higher motor areas (including the dominant PMd), instead of motor execution *per se* (i.e., the M1). It has been previously suggested (based on a DCM investigation; Hartwigsen et al., 2013) that functional connectivity between the pre-SMA and the dominant PMd is required for generating and sequencing motor plans for speech production. The age-related decline in remote connectivity within the higher motor areas (mainly including the dominant PMd) might be generalized to a neural basis of elderly's common difficulties in newly introduced delicate motor control task in accordance with information from the environment. Naturally, additional age comparisons are needed to fully support the possibility concerning the aging effect on the dominant PMd-related connectivity for planning of unfamiliar motor actions.

## Limitations and future directions

So far, the present SEM approach has yielded important conclusions concerning the significance and involvement of functional connectivity for bimanual finger coordination and its age-related decline. However, a possible limitation may lie in the selection of the specific ROIs. Although our ten ROIs within the motor association cortex convincingly follow Walsh et al.'s (2008) study, nevertheless, even within selected brain regions, subsequent choices in how to extract the fMRI data can have a substantial influence on the connectivity pattern (Bedenbender et al., 2011). Therefore, in order to verify the robustness of the functional connectivity patterns obtained in this study, further substantiation is necessary (preferably utilizing wide range of methodologies). Additionally, it is likely that other regions such as the basal ganglia, visual cortex and the cerebellum may also prove to be important candidates concerning functional connectivity for bimanual finger coordination (e.g., Battaglia-Mayer et al., 2001; Rizzolatti and Luppino, 2001; Nakai et al., 2003, 2005; Rowe et al., 2006; Grefkes et al., 2008; Hanakawa et al., 2008; Filimon, 2010; Leech et al., 2012; Zilverstand et al., 2014). Specifically, Aramaki et al.'s (2011) fMRI study on the phase-transition in young adults highlighted a potential role for the anterior putamen as a predictor of future instability for the anti-phase finger tapping. Witt et al.' (2008) meta-analysis utilizing SEM also suggests a diverse range of networks including cortical, subcortical and cerebellar regions. The current investigation focused on the motor association cortex in order to specifically highlight functional connectivity for bimanual finger coordination. However, naturally, more inclusive models are required to better understand the mechanisms involved in age-related decline in the coordination.

Another point of attention concerns the specific model construction procedure. SEM specifically permits multi-group analyses by comparing path coefficients across populations, on the basis of pairwise parameter comparisons (Arbuckle, 2012). However, this inevitably requires the same model to be applied to multiple groups. In the present study, different models had to be constructed for each of the two modes (in-phase and anti-phase) of the two groups since the connectivity patterns were considerably different across young and elderly groups. Due to the limitation of lacking pairwise parameter comparisons in our SEM assessment, we exclusively focused on strong path differences across groups, that is, the differences between significant paths at *p* < 0.001 and insignificant ones. It would be premature to compare other relatively small differences between path coefficients across models.

Additionally, it has been reported that neural networks in broader areas decline in age-related disorders such as MCI and AD patients (Stam et al., 2007; Bai et al., 2013). Therefore, further comparisons focusing on functional connectivity are required in order to better understand how the age-related decline in the connectivity suggested in the present study can be accelerated by any age-related disorders, or can be suppressed by any individual traits. Such comparisons could possibly lead to an effective index to allow for early diagnosis of the diseases.

## Conclusion

In order to explore the functional connectivity within the motor association cortex required for complex bimanual finger coordination (i.e., not simply the neural activation within each region itself), the present study utilized SEM to explore fMRI data obtained using a visually-guided bimanual finger movement task. This task contained a stable in-phase mode and a complex anti-phase mode and administered to both young and elderly age groups. Based on the present SEM exploration, two main conclusions were drawn: (1) the PMd in the dominant hemisphere acts as an intermediary to invoke intense intra- and inter-hemispheric connectivity with distant regions among the higher motor areas including the dominant S1 and the non-dominant SPL in order to achieve successful bimanual finger coordination, (2) the distant connectivity among the higher motor areas declines with aging whereas the local connectivity within the bilateral M1 is enhanced for the complex anti-phase mode. The latter may underlie the elderly's decreased performance in the complex anti-phase mode of the bimanual finger movement task.

### Conflict of interest statement

The authors declare that the research was conducted in the absence of any commercial or financial relationships that could be construed as a potential conflict of interest.
